# An Association Among Problematic Smartphone Use, Sleep Quality, Behavioral Engagement and Academic Achievements: A Multiple Mediation Model Based on Large-Scale Data

**DOI:** 10.3390/bs16050781

**Published:** 2026-05-15

**Authors:** Da Zhou, Shuting Liu, Jinqing Liu, Helin Li, Yue Ma

**Affiliations:** 1 Faculty of Education, Northeast Normal University, Changchun 130024, China; zhoud207@nenu.edu.cn (D.Z.); lihelin919@nenu.edu.cn (H.L.); 2 Examination Center of the Ministry of Public Security, Beijing 100741, China; gkz2025@163.com; 3 Education Department, College of Art and Science, University of Scranton, Scranton, PA 18510, USA; jinqing.liu@scranton.edu; 4 School of Education, Shanghai Jiao Tong University, Shanghai 200240, China

**Keywords:** problematic smartphone use, mathematics achievement, sleep quality, mathematics behavioral engagement

## Abstract

The purpose of this study is to investigate the mediating effects of sleep quality and mathematics behavioral engagement on the relationship between problematic smartphone use (PSU) and mathematics achievement while controlling for gender and socio-economic status (SES). A sample of 1645 fourth-grade students from southern China completed a math test and an online questionnaire assessing PSU, sleep quality, and mathematics behavioral engagement. Confirmatory factor analysis, descriptive statistics, Pearson’s correlation, and structural equation modeling were used in the data analysis. The findings showed that: (1) Boys exhibited higher levels of PSU than girls, and students from lower socioeconomic backgrounds were more prone to PSU. (2) PSU was negatively correlated with mathematics achievement. (3) Between PSU and mathematics achievement, mathematics behavioral engagement was a significant mediator. (4) Sleep quality and mathematics behavioral engagement played a significant sequential mediating role in the association between PSU and mathematics performance. This study focused on exploring the mechanisms at play in the relationship between PSU, sleep quality, behavioral engagement and mathematics achievement for primary school students.

## 1. Introduction

With the development of modern science and technology, smartphones continue to grow in popularity and have now become a necessary part of people’s lives around the world (Amez & Baert, 2020). According to the State of Mobile Internet Connectivity Report 2023 by the Groupe Special Mobile Association (GSMA), mobile Internet penetration continues to grow, with smartphones being the primary means of accessing the mobile Internet (GSMA, 2023). The International Telecommunication Union (ITU) reports in its Facts and Figures for 2023 that 78% of the world’s population aged ten and over will own a smartphone in 2023 (ITU, 2023). In China, the extent and intensity of Internet use among minors have increased significantly, with nearly 90 percent of minors owning their own Internet-enabled devices, predominantly smartphones (China Internet Network Information Center, 2023). While smartphones bring numerous benefits to minors, it is also important to be wary of their negative correlation with emotional health, social interactions and academic performance (Horwood & Anglim, 2019; Zhou et al., 2022). For instance, Zhou et al.’s study found that problematic smartphone use (PSU)—defined as compulsive and uncontrolled smartphone overuse that interferes with daily life, study and psychological well-being—is correlated with pupils’ anxiety symptoms, which are further associated with reduced learning interest and poorer academic performance. This finding highlights the negative correlation of smartphones with an individual’s emotion and learning, especially for the younger population.

Problematic smartphone use can manifest in various forms, often described in the literature as “smartphone addiction,” “problematic smartphone use,” “smartphone addiction proneness,” or “smartphone dependence” (L. Li et al., 2020). For the purposes of this study, we have adopted the term “problematic smartphone use” (PSU), as it more accurately reflects the nature of smartphone usage under some control. We define PSU as obsessive use of smartphones, characterized by behaviors that interfere with daily functioning or well-being (Kong et al., 2020; Zhou et al., 2021). By utilizing this terminology and definition, we aim to contribute to the growing body of literature on PSU among young populations while maintaining a balanced and age-appropriate perspective on the phenomenon.

Extant research indicates that many primary school students face academic performance challenges, with PSU emerging as a potential contributing factor (Amez & Baert, 2020; Kates et al., 2018; Sapci et al., 2021). The majority of studies in this area have concentrated on the relationship between PSU and general academic performance (Gi et al., 2016; Hawi & Samaha, 2016). However, with the exception of the team’s previous studies (Zhou et al., 2020b, 2021, 2022), limited attention has been given to the association between PSU and performance in specific academic subjects. Compared with general academic performance, mathematics achievement has stronger disciplinary specificity, logical rigor, and long-term predictive value for students’ subsequent academic and career development. Mathematics, given its gatekeeper role in educational systems globally (Gravemeijer et al., 2017), warrants particular attention. To address this evident research gap, the present study focuses on exploring the relationship between PSU and mathematics achievement. This specific subject-oriented design can capture the practical influence of PSU more precisely than adopting a general academic indicator alone. This study aims to contribute additional evidence from the Chinese context to the existing body of knowledge, thereby enriching the global understanding of this research field.

Extensive research demonstrates that PSU is negatively correlated with students’ sleep quality (S. Y. Kim et al., 2020) and behaviors (Zhou et al., 2022). Sleep quality is associated with students’ behavioral and cognitive performance (Curcio et al., 2006; Dewald et al., 2010). These findings suggest a plausible sequential association linking PSU to sleep quality, then to behavioral engagement, and further to mathematics academic performance. Based on previous relevant studies, it can be found that little attention has been paid to the chained mediating roles of sleep quality and behavioral engagement in the pathway between PSU and subject-specific academic achievement. Behavioral engagement is defined as the active participation of students in classroom activities (Fredricks & McColskey, 2012) and is one of the factors that is correlated with academic performance (Lei et al., 2018). In order to fill this research gap and provide empirical evidence for the hypothesized relationships, the present study explores the joint associational role of sleep quality and behavioral engagement in the linkage between PSU and mathematics achievement. As such, this study extends the existing research literature and offers implications for educational interventions aimed at improving students’ mathematics achievement.

The 3P model, derived from Dunkin and Biddle’s (1974) “presage-process-product” framework, offers a dynamic approach to understanding the student learning process (Biggs, 1993). This model posits that learning is a complex interplay of presage, process, and product stages, where presage encompasses students’ pre-existing characteristics and learning context, process involves learning conditions and engagement, and product refers to learning outcomes (Biggs et al., 2001). In line with the theoretical connotation of the 3P model, PSU and sleep quality are classified as presage factors, because both reflect students’ relatively stable behavioral tendencies and physiological–psychological baseline status formed prior to formal learning. By comparison, mathematics behavioral engagement naturally fits the definition of a process factor, as it occurs in real-time learning activities and acts as the core intermediate pathway linking pre-learning conditions to final academic outcomes. The 3P model conceptualizes learning as a dynamic system in which student prior factors, the learning process, and outcomes interact reciprocally. In this system, predetermined factors typically influence products indirectly through the mediating pathway of process factors (M. H. Lee et al., 2020). This theoretical framework has been empirically validated in educational research, with several studies employing it as a foundation for their theoretical constructs. For instance, Guo et al. (2022) developed a structural equation model based on the 3P framework, examining the relationships between perception of the learning environment (presage), engagement (process), and learning outcomes (product). In this model, engagement served as a mediating process factor. Similarly, H. Li et al. (2023) utilized the 3P model to investigate the direct effects of information literacy self-directed learning ability (presage) and learning emotions (process) on high school students’ online learning participation (product) while also exploring the mediating role of process factors.

Building upon this theoretical foundation, the present study constructs a chain mediation model to explore the associational patterns among PSU and sleep quality (presage factors), behavioral engagement (process factor), and students’ mathematics academic performance (product). This model hypothesizes that PSU and sleep quality, as presage factors, are closely associated with students’ mathematics behavioral engagement in the learning process, which is further linked to their academic performance. By targeting the underexplored chained mediation pathway within the 3P theoretical framework, this study extends the 3P theory and enhances the model’s generalizability. Furthermore, it aims to advance both theoretical understanding and practical applications regarding the complex interplay between PSU and academic achievement. The main contributions of this study are twofold. First, it shifts the research focus from general academic performance to mathematics-specific achievement, yielding more discipline-targeted empirical evidence. Second, it integrates PSU, sleep quality, and behavioral engagement into a unified 3P framework to clarify their sequential chained mediating pathway, remedying the limitation of simple pairwise comparisons in existing studies. This research broadens the applicability of the 3P model in primary mathematics education, deepens theoretical understanding of the link between PSU and subject-specific learning outcomes, and provides practical implications for targeted educational intervention and instructional practice.

## 2. Literature Review and Theoretical Model

### 2.1. The Relationship Between PSU and Academic Achievement

Based on the 3P model, it is known that students’ existing states and situations are associated with learning outcomes. The student’s PSU, then, is the presage in the 3P model and is correlated with their academic achievement. Extensive research supports the negative relationship between PSU and academic achievement (Amez & Baert, 2020; Hawi & Samaha, 2016; Kates et al., 2018; Paterna et al., 2024). For instance, the findings of meta-analytic research have reported a small-sized negative relationship between both PSU and academic achievement (i.e., r = −0.12) (Kates et al., 2018). Another meta-analysis study also found a small effect size (r = −0.11), which was more pronounced in samples of elementary and middle school students (Paterna et al., 2024). It is important to note that these samples were taken from previous research conducted by our team. These findings collectively highlight the potential negative correlation between PSU and students’ academic performance. However, while most existing studies focus on the relationship between PSU and the academic performance of college and high school students (Paterna et al., 2024), few have examined elementary school students. Since there is limited knowledge about pupils’ smartphone usage in China, this study aims to further investigate pupils from other regions of China to validate the effectiveness of the proposed model in linking PSU and mathematics achievement. This will ensure a broader generalization of the results and provide more comprehensive Chinese evidence.

### 2.2. The Mediating Role of Behavioral Engagement

Research has consistently demonstrated a significant correlational link between students’ cognitive, emotional, and behavioral engagement and their mathematics achievement (Lei et al., 2018). Within the 3P model, behavioral engagement, functioning as a learning process, is a multidimensional construct that is associated with academic outcomes (M. T. Wang et al., 2016). Behavioral engagement is positively correlated with students’ achievement in mathematics (J. Chen et al., 2020; L. Wang et al., 2022). For instance, some scholars have demonstrated an association between behavioral engagement and achievement by using the frequency of hand-raising to represent students’ behavioral engagement in the classroom (Böheim et al., 2020).

Although we would like to review the link between PSU and learning engagement, few studies focus on this association. However, we found that some scholars have studied the negative correlation between Internet addiction and college students’ learning engagement, in which learning engagement encompasses behavioral, affective, and cognitive aspects (R. Q. Sun et al., 2023; Zhang et al., 2018). And there is compelling evidence from a six-month longitudinal study in China showing a negative correlation between Internet addiction and students’ engagement in learning (Zhang et al., 2018).

While existing research predominantly focuses on the direct correlational link between learning engagement and academic achievement and the negative correlation between Internet addiction and learning engagement, there remains a need for more nuanced exploration. As Internet access devices include not only smartphones but also computers and tablets, findings on Internet addiction cannot be directly generalized to PSU. Consequently, it is necessary to examine the role of behavioral engagement in the relationship between PSU and academic achievement for enriching current studies.

### 2.3. The Mediating Role of Sleep Quality in Two Links

More and more elementary school students are using smartphones, and while they derive convenience and enjoyment from them, they also face a range of potential health risks, such as decreased sleep quality, increased anxiety, and rising obesity rates (J. Yang et al., 2020). Among these risks, the most direct and significant correlation is the decline in sleep quality (Lane et al., 2021). Many elementary school students often become engrossed in smartphone use after school, engaging in activities such as online gaming with peers, social communication, watching short videos, or television programs. Prolonged immersion in the smartphone world is correlated with reduced children’s rest time, increased cognitive load, emotional disturbance, and elevated physiological arousal levels, which are associated with poorer sleep quality (X. Xie et al., 2018). Notably, a negative correlation between PSU and sleep quality has been extensively validated in adult populations (Chang et al., 2022; Kaya et al., 2021; Sohn et al., 2021). Given that children are in a critical period of rapid physical and mental development, further validation of this issue among the pediatric population and implementation of appropriate strategies are of paramount importance.

Furthermore, within the 3P model, presage factors such as sleep quality are associated with students’ behavioral engagement and cognitive performance, which serve as learning processes and outcomes, respectively. Additionally, the relationship among students’ sleep quality, behavioral engagement, and academic performance is supported by attentional resources theory (Kahneman, 1973) and working memory theory (Baddeley, 1992).

From the perspective of attentional resources theory, when new stimuli are presented to individuals whose cognitive resources have been depleted, these novel stimuli are unlikely to be processed effectively (Kahneman, 1973). Individuals experiencing poor sleep quality tend to have dispersed or insufficient attentional resources (W. Xie et al., 2019). Consequently, inadequate sleep in adolescents is associated with a wide array of adverse outcomes, ranging from compromised mental and physical health to behavioral problems. A study that surveyed university students in Australia and Hong Kong found that poor sleep quality is correlated with their academic engagement (Nelson, 2018; Ng et al., 2022). Studies of middle school students have also consistently concluded that poor sleep quality is negatively correlated with academic engagement. Bastian and Fuller (2023) found that middle school students with late school start times tended to have high levels of academic engagement, suggesting a positive correlation between extended sleep time and students’ academic engagement.

From the perspective of working memory theory, an individual’s working memory is a system or series of systems used to temporarily store and process information while solving cognitive tasks, and it plays a crucial role in accomplishing complex mental activities, including verbal comprehension, decision-making processes, and mental arithmetic (Baddeley, 1992). Notably, sleep deprivation is correlated with impaired working memory function, which is associated with students’ cognitive performance (Durmer & Dinges, 2005). Substantial evidence indicates that sleep is critical for cognitive function and plays a vital role in memory consolidation and academic performance (Fonseca & Genzel, 2020; Rathakrishnan et al., 2021). Sleep quality has a significant correlation with students’ neurological state, and studies have shown a positive correlation between improved sleep quality and the academic performance of college students (Okano et al., 2019).

Synthesizing the above literature, a consistent conclusion has been formed: PSU is adversely correlated with sleep quality, and sleep quality further associates with students’ behavioral engagement and academic performance based on attentional resources and working memory theories. However, most existing empirical evidence is derived from adult and middle school student groups. Systematic research that simultaneously incorporates PSU, sleep quality, behavioral engagement and academic performance to construct a chained mechanism among elementary school students is still underexplored, leaving an important research gap in younger populations.

### 2.4. The Gender and SES as Control Factors

In educational research, gender and socio-economic status (SES) are important factors associated with students’ academic performance (Alordiah et al., 2015; Ewumi, 2012). Therefore, this study also focuses on the gender and SES of the students. Studies have shown that women tend to use the Internet primarily for social interactions (Peris et al., 2020), while men primarily engage in online gaming (Hassan et al., 2020). Albursan et al. (2019) found a correlation between women and a higher risk of smartphone addiction than men. Consistent with these data, in the study by Mari et al. (2023), women scored higher than men on the dimension of obsession with cell phones, which is associated with women often needing to check their cell phones to make sure that they have new information. While some studies have the opposite finding, some scholars found that the incidence of Internet addiction is higher among boys than girls by surveying Korean teenagers (Ha & Hwang, 2014). According to the results of a sample survey in China, the percentage of Internet addicts was significantly higher among males (14.8%) than females (7%), and the percentage of Internet addiction among elementary school students (11.5%) was comparable to that of middle school students (11.9%) (Y. Li et al., 2014). To reconcile these divergent results, further investigation into gender differences in PSU using a larger, more representative sample is warranted. SES is an important factor associated with Internet addiction (X. Sun et al., 2021; Urbanova et al., 2019). For example, one study found that adolescents with lower socioeconomic status had a higher probability of Internet addiction (W. Chen et al., 2025). However, relevant research at the elementary school level remains relatively limited, so there is an urgent need to explore the relationship between PSU and SES in specific student populations.

Given the significant correlation of both gender and socioeconomic status with smartphone use patterns and academic performance, it is crucial to account for these factors when examining the relationships among other variables of interest. Therefore, in this study, we control for the correlations of gender and SES to isolate and more accurately assess the associations of other variables under investigation.

### 2.5. The Theoretical Model and Research Hypotheses

The sequential mediation structure of the present model is theoretically grounded and empirically justified. First, according to attentional resources theory and working memory theory, sleep quality serves as a fundamental physiological and psychological prerequisite that determines students’ cognitive energy and attentional state, which logically precedes and further shapes their classroom behavioral engagement. Therefore, it is theoretically reasonable to place sleep quality prior to behavioral engagement in the chain pathway. Second, following the presage-process-product logic of the 3P model, PSU and sleep quality are inherent presage factors, behavioral engagement acts as a core process factor, and mathematics achievement represents the final product outcome. This hierarchical theoretical framework naturally supports the constructed chain mediation pathway rather than alternative model specifications. This model structure is therefore not arbitrarily designed but derived from theoretical connotations and existing empirical evidence. The proposed multiple mediation model, along with the corresponding research hypotheses, are presented as follows.

**H1.**  *Gender and SES are correlated with being problematic smartphone users.*

**H2.**  *PSU is negatively correlated with mathematics achievement.*

**H3.**  *Mathematics behavioral engagement plays a mediating role in the relationship between PSU and mathematics achievement.*

**H4.**  *Sleep quality mediates the relationship between PSU and mathematics achievement.*

**H5.**  *Sleep quality and then mathematics behavioral engagement serially mediate the relationship between PSU and mathematics achievement.*

## 3. Methods

### 3.1. Participants

The data for this study come from a large-scale education assessment program in China, in which our team was actively involved. A two-stage stratified sample was used. In 2022, one city in southern China was randomly selected following standard stratified sampling protocols and the regulations of the local education bureau, rather than being deliberately chosen for geographical preference, and a total of 16 schools were randomly sampled from this city. Subsequently, fourth-grade students from each school were randomly assigned to participate in the study, yielding a total sample of 1673 students. The final sample comprised 1645 students (the response rate of participants was 98.3% > 90%), of whom 873 (53.1%) were male and 772 (46.9%) were female. The participants ranged in age from 9 to 11 years, with a mean age of 10 years.

### 3.2. Measures

The measurement instruments employed in this study comprised scales assessing problematic smartphone use, sleep quality, and mathematics behavioral engagement. These scales were adopted from established research in their respective fields. Additionally, to evaluate students’ mathematics performance, we utilized standardized tests developed by the Regional Education Assessment Project (REAP). The subsequent sections provide a detailed description of each instrument.

#### 3.2.1. Demographic Information

Demographic data were collected from participants, including gender and SES. Gender was coded dichotomously, with 1 representing male and 2 representing female. The SES was constructed using three key components: family possessions, parental occupational status, and parental educational attainment. Following the methodology outlined by the OECD (OECD, 2017) and consistent with previous research (Zhou et al., 2020a), we employed principal component analysis to compute a comprehensive SES score. The score is positively correlated with socioeconomic status; higher scores indicate a higher SES.

#### 3.2.2. Mathematics Achievement Test

The REAP team developed a comprehensive mathematics achievement test aligned with the Full-time Compulsory Education Mathematics Curriculum Standards (2011 edition). This assessment instrument comprises 16 items, each designed to evaluate specific content and cognitive domains. The content areas assessed are based on three content domains: numbers and algebra, measurement and geometry, and statistics and probability. The cognitive dimensions evaluated include: knowing, understanding, and applying. The test is based on the curriculum standards and consists of 10 multiple-choice questions and 6 subjective questions.

Several researchers in the REAP group have published descriptions of academic performance tests in mathematics (Liu et al., 2019; Zhou et al., 2021). In this study, the Item Response Theory (IRT) method was used, which involves calculating item parameters using ConQuest software v5. The Cronbach’s alpha for the mathematical test was 0.86, indicating good agreement (Cortina, 1993).

#### 3.2.3. Problematic Smartphone Use

The PSU scale was from the Smartphone Addiction Proneness Scale for Youth (D. Kim et al., 2014). A part of the items was chosen based on their superior index values and highest factor loadings. The PSU scale in this study comprised five items, including statements such as “Even though I know I should not spend too much time using my phone, I will keep using it” and “I am used to spending much time on my phone.” Participants responded using a four-point Likert scale (1 = strongly disagree to 4 = strongly agree). The scale has demonstrated high reliability and validity in previous studies (Lai et al., 2020; L. Li et al., 2016). The cumulative score indicates an individual propensity for PSU, with higher scores suggesting a higher inclination towards problematic smartphone usage. The scale’s model fit indices were found to be satisfactory ($\chi^{2}$ (5, 1645) = 160.558, *p* < 0.001, CFI = 0.964, TLI = 0.927, RMSEA = 0.038, and SRMR = 0.027), meeting the criteria proposed by Hu and Bentler (1999). Furthermore, the scale’s internal consistency was robust, with a McDonald’s omega of 0.88, exceeding the 0.80 threshold considered acceptable in psychological research (Hayes & Coutts, 2020; Watkins, 2017).

#### 3.2.4. Sleep Quality

The Sleep Quality Questionnaire is derived from the Sleep Quality Scale (SQS), a comprehensive and effective tool for evaluating sleep quality across diverse patient groups and research populations (Yi et al., 2006). This scale comprises three items (e.g., “I am satisfied with my sleep”; “I felt refreshed upon waking”). Participants were asked to respond on a four-point Likert scale based on their sleep experiences, where 1 = rarely (0–3 times per month), 2 = sometimes (1–2 times per month), 3 = often (3–5 times per month), and 4 = always (6–7 times per month). Higher scores indicate better sleep quality. It should be noted that the SQS, as a one-factor model consisting of three items, has zero degrees of freedom, meaning it is a just-identified (saturated) model. By definition, a saturated model guarantees perfect fit; thus, the reported model fit indices ($\chi^{2}$ (0, 1645) = 0, *p* = 0.000, CFI = 1.000, TLI = 1.000, RMSEA = 0.000, and SRMR = 0.000) do not provide empirical evidence of good model fit, which constitutes a limitation of this measurement. To further ensure the scale’s quality, the factor loadings of its three items were calculated as 0.679, 0.878, and 0.710, respectively. Additionally, the scale’s internal consistency reliability, as measured by McDonald’s omega, was 0.80, indicating strong reliability.

#### 3.2.5. Mathematics Behavioral Engagement

The Mathematics Behavioral Engagement Scale is adapted from the Math Engagement Scale developed by M. T. Wang et al. (2016). Mathematics behavioral engagement refers to students’ positive behaviors during math classroom activities, as opposed to disruptive behaviors. These positive behaviors include attention, concentration, adherence to math class rules, and completion of math homework (Fredricks & McColskey, 2012). The scale comprises four self-report items (e.g., “I complete my math homework on time”; “When studying math, I stay focused”) rated on a five-point Likert scale (1 = strongly disagree to 5 = strongly agree). Higher total scores indicate greater levels of mathematics behavioral engagement. The scale demonstrated acceptable model fit indices: $\chi^{2}$ (2, 1645) = 2.388, *p* < 0.001, CFI = 1.000, TLI = 1.000, RMSEA = 0.011, and SRMR = 0.002. The internal consistency reliability of the scale, estimated using McDonald’s omega, was notably high at 0.93.

### 3.3. Procedure and Analysis

Students participated in a comprehensive assessment process consisting of two parts. First, they completed a 70-min paper-based mathematics achievement test. Following this, they were invited to fill out an online questionnaire, which took approximately 20 min to complete. This questionnaire included measures of PSU, sleep quality, and mathematics behavioral engagement. All participants received identical tests and instructions to ensure consistency. A team of graduate students specializing in mathematics education was trained to grade the math achievement tests using standardized scoring criteria. These graders were required to achieve a high inter-rater reliability (95% agreement) before being authorized to conduct formal coding of the tests. To ensure the highest standards of data collection and analysis, REAP contracted professional companies to administer both the tests and questionnaires. This approach guaranteed the integrity and reliability of the data gathered for subsequent analysis.

In the analytical process, we first conducted confirmatory factor analysis (CFA) to test whether the items in the construct aligned with our conceptual understanding (McInerney et al., 2012; Zhou et al., 2020b). We then performed descriptive statistical analysis to examine the distribution of all variables, followed by Pearson’s correlation analysis to determine the correlation coefficients between variables (Zhou et al., 2021). The results revealed significant correlations between gender, SES, and other variables. Consequently, we controlled for these two variables in the subsequent analyses.

**Figure 1:**
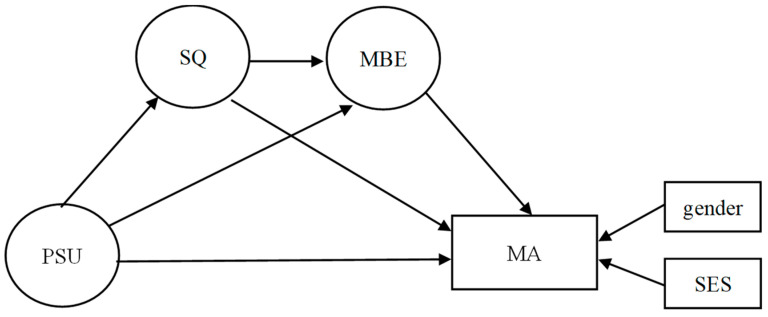
The proposed multiple mediation model. Notes. PSU = Problematic Smartphone Use; SQ = Sleep Quality; MBE = Mathematics Behavioral Engagement; MA = Mathematics Achievement; SES = Socio-economic Status.

We employed a two-step approach to investigate the mediating roles of sleep quality and mathematics behavioral engagement in the relationship between PSU and mathematics achievement. The first procedure was to explore the direct relationship between PSU and mathematics achievement. The second procedure was to examine the indirect effects of sleep quality and mathematics behavioral engagement in a multiple mediation model (Figure 1). We then randomly selected 5000 repeated resamples with replacement from the original data and estimated 95% bias-corrected confidence intervals for both direct and indirect effects. The effect was considered significant if the confidence intervals did not include 0 (Erceg-Hurn & Mirosevich, 2008).

Data analysis was conducted using SPSS 22.0 and Mplus 7.0 software. To ensure the validity of this study, we performed a common method bias test (Podsakoff et al., 2003). We employed Harman’s single-factor method, one of the most widely adopted techniques for examining common method bias (Tehseen et al., 2017). Exploratory factor analysis can indicate no serious methodological bias if the first of the three factors has an explanatory rate of less than 40% (Kong et al., 2020). Considering the inherent limitation that Harman’s single-factor test has relatively low sensitivity when used independently, we further adopted the common latent factor (CLF) approach for supplementary verification. All questionnaire items were included to extract a global common latent factor, and the corresponding factor score was incorporated as a control variable into subsequent statistical analysis. The results showed that the regression coefficients (∆β=0.006<0.1) remained stable after controlling for the common latent factor, confirming that common method bias was not a prominent issue in this study.

## 4. Results

### 4.1. Preliminary and Descriptive Analyses

To assess the construct validity of the questionnaire, which included three interrelated scales measuring PSU, sleep quality, and mathematics behavioral engagement, we conducted CFA. Preliminary results revealed a clear three-factor structure, with all factor loadings exceeding 0.60 (see Table 1). The model fit indices were as follows: $\chi^{2}$ (51, n = 1645) = 225.676, *p* < 0.001; RMSEA = 0.046; CFI = 0.985; TLI = 0.980; SRMR = 0.021. All of them met the criteria proposed by Hu and Bentler (1999).

Furthermore, discriminant validity of the three latent constructs was verified following the Fornell-Larcker criterion. The square root of AVE for PSU was 0.777, which was greater than the absolute values of its correlation coefficients with sleep quality (r = −0.245) and mathematics behavioral engagement (r = −0.317). The square root of AVE for sleep quality was 0.761, exceeding its correlation coefficients with PSU (r = −0.245) and mathematics behavioral engagement (r = 0.217). The square root of AVE for mathematics behavioral engagement was 0.878, which was higher than its correlation coefficients with PSU (r = −0.317) and sleep quality (r = 0.217). All latent constructs satisfied the Fornell-Larcker requirement, confirming adequate discriminant validity and empirical distinction among PSU, sleep quality, and mathematics behavioral engagement.

As presented in Table 2, significant correlations were observed between gender and SES and all other variables. Male students demonstrated a higher propensity for PSU compared to their female counterparts (r = −0.143, *p* < 0.01). Students from higher SES backgrounds exhibited fewer smartphone-related issues (r = −0.178, *p* < 0.01), better sleep quality (r = 0.129, *p* < 0.01), greater mathematics behavioral engagement (r = 0.206, *p* < 0.01), and higher academic grades (r = 0.330, *p* < 0.01). Consequently, gender and SES were controlled for in subsequent mediation analyses.

These findings indicated that PSU was negatively correlated with sleep quality (r = −0.245, *p* < 0.01), mathematics behavioral engagement (r = −0.317, *p* < 0.01), and academic achievement (r = −0.184, *p* < 0.01). These results suggest that students who engage in PSU tend to experience poorer sleep quality, lower mathematics behavioral engagement, and reduced academic performance.

### 4.2. Testing for the Multiple Mediation Model

Prior to analyzing the multiple mediation model, we examined the direct effect of PSU on mathematics achievement, while controlling for both gender and SES. The direct effect model yielded marginal fit indices: $\chi^{2}$ (19, 1645) = 246.208, *p* < 0.001, CFI = 0.949, TLI = 0.928, RMSEA (90% CI) = 0.085, SRMR = 0.059. These values did not fully meet the strict cutoff criteria proposed by Hu and Bentler (1999). The marginal fit observed in the present study can be mainly explained by model parsimony. Although gender and SES were included as control variables, the model contained only one core independent variable and one outcome variable. To preserve theoretical parsimony, no additional predictors, mediators, or correlated residual paths were added merely to improve model fit, which reasonably accounts for the marginally acceptable fit statistics. Given the suboptimal model fit, a supplementary manifest-level analysis ($R^{2}$ = 0.12) was further conducted as a robustness check, which verified the stability of the direct association based on raw observed scores. Results indicated that, after controlling for gender and SES, PSU had a significant negative direct effect on mathematics scores (β = −0.178, *p* < 0.001), suggesting that higher levels of PSU were associated with poorer mathematics achievement in elementary school students.

**Figure 2:**
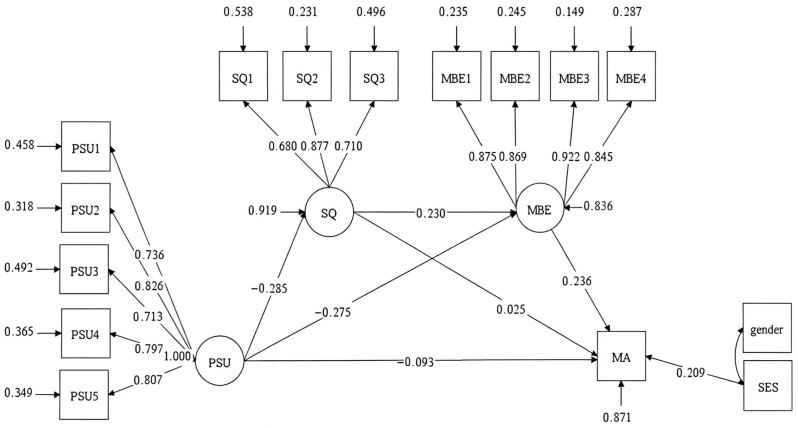
The standardized results of the multiple mediation model. Notes. PSU = Problematic Smartphone Use; SQ = Sleep Quality; MBE = Mathematics Behavioral Engagement; MA = Mathematics Achievement; SES = Social economic status.

We then tested a multiple mediation model, proposing that sleep quality and mathematics behavioral engagement mediated the relationship between PSU and mathematics achievement (Figure 2), while controlling for gender and SES. The model demonstrated acceptable fit: $\chi^{2}$ (84, 1645) = 357.865, *p* < 0.001, CFI = 0.977, TLI = 0.972, RMSEA (90% CI) = 0.045, and SRMR = 0.043. To assess the statistical significance of the indirect paths, we employed a bias-corrected bootstrap test.

As presented in Table 3, after controlling for gender and SES, the mediating effect of mathematics behavioral engagement and the sequential mediating effect of sleep quality through mathematics behavioral engagement were significant, as indicated by 95% confidence intervals not including zero. However, the mediating effect of sleep quality alone was not significant. Specifically, indirect Path 1 (PSU → sleep quality → mathematics achievement), which reflects the sole mediating role of sleep quality, accounted for 3.93% of the total effect and was statistically non-significant. By contrast, indirect Path 2 (PSU → mathematics behavioral engagement → mathematics achievement) explained 36.52% of the total effect and reached statistical significance. The sequential mediation Path 3 (PSU → sleep quality → mathematics behavioral engagement → mathematics achievement) accounted for 8.43% of the total effect and was also statistically significant. Collectively, these two significant indirect paths jointly explained 44.95% of the total effect.

Although the study’s large sample size (n = 1645) increases the likelihood that even small effects may reach statistical significance, the effect size analysis using the proportion of total effect mediated (PEM) provides a more nuanced assessment of practical importance. The significant indirect paths together explained nearly half (44.95%) of the total effect, demonstrating that the observed mediation mechanisms are not merely statistically significant but also substantively meaningful.

## 5. Discussion

This study made several significant contributions to the field of mathematics education research and practice. We will discuss each of these contributions in turn.

Firstly, the results supported H1, indicating that both gender and SES were significantly correlated with PSU. Specifically, these findings reveal that boys are more susceptible to PSU than girls, which supported H1. This result is different from those of some previous studies (Albursan et al., 2019; S. Y. Yang et al., 2018) but is consistent with previous studies by our team (Zhou et al., 2022). In addition, in surveys of Korean teenagers and other Chinese elementary school students, the results also showed that boys were more likely to be addicted to the Internet than girls (Ha & Hwang, 2014; Y. Li et al., 2014). The reasons why boys are more susceptible to PSU than girls in our sample are that boys seem passionate about playing games and watching videos online, among other things, but girls seem to care more about their relationships with their peers at the elementary school level (Gentina & Rowe, 2020). This finding emphasizes the importance of considering specific gender groups when studying PSU and highlights the need for targeted interventions for different gender group. Furthermore, findings suggest that students with lower SES are more likely to be affected by PSU, consistent with existing research (W. Chen et al., 2025; X. Sun et al., 2021; Urbanova et al., 2019). Students with low SES may receive relatively insufficient parental attention, which is linked to a greater tendency to rely on smartphones for entertainment and social fulfillment and correlates with higher levels of PSU. Although parents of low-SES students are generally aware of the potential harms of PSU, they often lack effective strategies to guide or manage their children’s smartphone use behavior (Zhou et al., 2022). Findings expand our understanding of SES difference in PSU among elementary school students and provide evidence for effective interventions.

Second, this study shows that PSU is negatively correlated with students’ mathematical achievements, which supported H2. The results of the study are also consistent with the 3P model. When students overuse their smartphones, they tend to be distracted from their studies and have difficulty concentrating, which is associated with lower academic performance (Gi et al., 2016). Furthermore, these students frequently experience interruptions from other applications during learning and find it challenging to regulate their smartphone use for educational purposes (J. Lee et al., 2015). By concentrating on elementary school students, this study provides Chinese evidence of the adverse relationship between PSU and mathematical achievement. This extends previous findings on PSU’s detrimental effects on general academic performance in middle school and college students (Amez & Baert, 2020; Hawi & Samaha, 2016; Kates et al., 2018).

Third, the results supported H3, indicating that mathematics behavioral engagement mediates the relationship between PSU and students’ mathematical achievement--a novel finding in the field. This can be elucidated through the 3P model, which posits that problematic smartphone use, as a presage factor, is negatively correlated with the process factor of mathematics behavioral engagement; meanwhile, mathematics behavioral engagement is associated with the outcome of mathematical achievement (Zhen et al., 2020). More specifically, behavioral engagement refers to students’ readiness to put in effort towards learning by utilizing cognitive, metacognitive, and voluntary strategies to improve their comprehension (Wong et al., 2003). Previous studies have established a significant link between PSU and behavioral engagement (R. Q. Sun et al., 2023; Zhen et al., 2020), and the association between PSU and subject-specific behavioral engagement was further justified in this study. In addition, previous studies have also found the link between behavioral engagement and academic achievement (Asif et al., 2020). For instance, diligence, a measure of behavioral engagement, is positively correlated with academic performance, as diligent students tend to think more and complete more tasks (Gasevic et al., 2017). These findings extend this knowledge by demonstrating that PSU is associated with subject-specific behavioral engagement, which in turn correlates with mathematical achievement. This insight helps identify critical pathways through which PSU relates to mathematical performance, offering valuable implications for educational interventions and policies.

The fourth finding shows that the path of sleep quality as a mediating variable was not significant, so H4 was not supported. However, the path of PSU-sleep quality-mathematics behavioral engagement-mathematics achievement was found to be significant, which supported H5. With the increasing prevalence of smartphone use, it has become harder for individuals to disconnect from their devices before bedtime. This habit has been linked to poorer sleep quality, including a decrease in rapid eye movement (REM) sleep, slow wave sleep, and overall sleep efficiency (Sohn et al., 2021; Dworak et al., 2007). Scholars suggest that the screen light suppresses the secretion of melatonin and delays sleep onset (Wood et al., 2006). When students’ sleep quality is poorer, it is negatively correlated with their academic performance, as Dewald et al. (2010) demonstrated through a meta-analysis of 16 studies of children and adolescents. Similarly, Astill et al. (2012) conducted a comprehensive meta-analysis of published data and found significant positive associations between sleep duration and executive function, academic performance, and multi-domain cognitive function. Research on sleep deprivation has shown that sleep restriction is negatively associated with performance in cognitive tests, including motor skills, language learning, and abstract thinking (Durmer & Dinges, 2005). These observed associations in children and adolescents are comparable to those in adults (Lo et al., 2016). Despite existing studies indicating that PSU is correlated with sleep quality and that sleep quality is associated with general academic achievement, this study shows that sleep quality did not directly mediate the relationship between PSU and mathematical achievement. A plausible explanation for elementary school student group is that sleep quality is not simply linked to academic achievement in an independent mediating pathway with PSU; instead, it is involved in a sequential mediation chain together with other behavioral factors such as students’ mathematical behavioral engagement, which constitutes a new empirical finding.

The relationship between sleep quality and mathematics behavioral engagement can be supported by the attentional resources theory and the 3P model, aligning with existing studies (Durmer & Dinges,2005; Ng et al., 2022; Nelson, 2018). From the perspective of these theories, insufficient sleep is closely associated with students’ mathematics behavioral engagement, alongside limited attentional resources and compromised working memory. Relevant empirical studies also support such theoretical rationales. For example, prior research indicates that sleep quality is negatively correlated with students’ academic engagement (Ng et al., 2022; Nelson, 2018), and sleep deficiency correlates with undesirable learning behaviors, which are further linked to lower academic performance (Gilbert & Weaver, 2010). Based on the above arguments, this study found that the association between sleep quality and behavioral factors is stronger than its correlation with academic performance among elementary school students.

## 6. Conclusions and Limitation

In conclusion, this study yields several meaningful findings and offers certain theoretical and practical implications. First, it preliminarily verifies the applicability of the 3P model within an Eastern cultural context through a well-fitted structural equation model, which may help enrich and extend the contextual adaptation of the model. Second, it explores multiple mediating pathways linking PSU, sleep quality, mathematics behavioral engagement, and mathematics achievement, which helps further clarify the associational relationships among these variables. Third, this study focuses on Chinese primary school students—a group less discussed in existing relevant literature—and adopts a relatively large sample, which can provide supplementary evidence for understanding smartphone use and academic performance among child populations beyond Western contexts. Fourth, the present findings also underline the important role of mathematics behavioral engagement, suggesting that educators may pay close attention to students’ learning engagement in mathematics instruction. This study may offer empirical references and practical enlightenment for routine educational practice.

The study has several limitations. First, this study collected data in 2022 within a post-pandemic context. The prevalence of online learning and increased exposure to digital devices during this period may have influenced students’ overall levels of PSU, sleep quality, and behavioral engagement. Nevertheless, although the post-pandemic setting may affect the absolute mean scores of these variables, it is unlikely to alter their core correlational associations and chained mediational mechanisms, which are theoretically grounded and empirically supported by the existing literature. Future studies could conduct sampling across different time periods to further validate the generalizability of the present findings.

Second, the cross-sectional design limits causal inferences, although it successfully identified associations among PSU, sleep quality, mathematics behavioral engagement, and achievement. Additionally, potential self-report bias may exist due to the sensitive nature of these variables, despite efforts to minimize bias through clear survey instructions. Future research should employ longitudinal designs and incorporate multidimensional data collection methods to establish causality and enhance reliability.

Third, all participants were recruited from only one city in southern China. Although the two-stage stratified random sampling procedure was rigorous and the sample size was sufficient, the single-city scope may restrict the cross-regional generalizability of the findings. Future research could expand sampling to multiple regions and different tiers of cities to verify and enrich the applicability of the results. In addition, the exclusive focus on fourth graders may limit generalizability; future studies should include diverse age groups to broaden the applicability of findings.

## Figures and Tables

**Table 1:** Factor loadings of problematic smartphone use, sleep quality, and mathematics behavioral engagement were analyzed based on a complete measurement CFA.

Item	PSU	SQ	MBE
Problematic Smartphone Use (PSU)			
PSU1	0.736		
PSU2	0.825		
PSU3	0.713		
PSU4	0.797		
PSU5	0.807		
Sleep Quality (SQ)			
SQ1		0.679	
SQ2		0.878	
SQ3		0.710	
Mathematics Behavioral Engagement (MBE)			
MBE1			0.874
MBE2			0.870
MBE3			0.922
MBE4			0.845

**Table 2:** Mean, standard deviations, and correlations among all the variables.

Variables	Gender	SES	PSU	SQ	MBE	MA
Gender	1					
SES	−0.010	1				
PSU	−0.143 **	−0.178 **	1			
SQ	−0.057 *	0.129 **	−0.245 **	1		
MBE	0.076 **	0.206 **	−0.317 **	0.271 **	1	
MA	0.024	0.330 **	−0.184 **	0.131 **	0.290 **	1
Mean	-	-	1.770	2.901	4.269	542.189
Standard Deviation	-	-	0.813	1.011	0.948	73.203

* *p* < 0.05, ** *p* < 0.01.

**Table 3:** Bias-corrected bootstrap test on the mediating effects.

Path	Standardized β	95% CI	
		Low	High
Path 1 PSU-SQ-MA	−0.007	−0.023	0.009
Path 2 PSU-MBE-MA	−0.065 ***	−0.091	−0.044
Path 3 PSU-SQ-MBE-MA	−0.015 ***	−0.023	−0.010
Indirect effects	−0.087 ***	−0.118	−0.063
Total effect	−0.178		

*** *p* < 0.001.

## Data Availability

The data supporting the findings of this study are not publicly available due to privacy and confidentiality restrictions to protect participants’ sensitive information.

## References

[B1-behavsci-16-00781] Albursan I. S., Al Qudah M. F., Dutton E., Hassan E. M. A. H., Bakhiet S. F. A., Alfnan A. A., Aljomaa Hammad H. I. (2019). National, sex and academic discipline difference in smartphone addiction: A study of students in Jordan, Saudi Arabia, Yemen and Sudan. Community Mental Health Journal.

[B2-behavsci-16-00781] Alordiah C. O., Akpadaka G., Oviogbodu C. O. (2015). The influence of gender, school location and socio-economic status on students’ academic achievement in mathematics. Journal of Education and Practice.

[B3-behavsci-16-00781] Amez S., Baert S. (2020). Smartphone use and academic performance: A literature review. International Journal of Educational Research.

[B4-behavsci-16-00781] Asif M., Thomas G., Awan M. U., Muhammad D. A. (2020). Enhancing student engagement through heterogeneous pedagogical approaches: Action research in a university level course in Saudi Arabia. International Journal of Educational Management.

[B5-behavsci-16-00781] Astill R. G., Van der Heijden K. B., Van IJzendoorn M. H., Van Someren E. J. (2012). Sleep, cognition, and behavioral problems in school-age children: A century of research meta-analyzed. Psychological Bulletin.

[B6-behavsci-16-00781] Baddeley A. (1992). Working memory. Science.

[B7-behavsci-16-00781] Bastian K. C., Fuller S. C. (2023). Late, but right on time? School start times and middle-grade students’ engagement and achievement outcomes in North Carolina. American Journal of Education.

[B8-behavsci-16-00781] Biggs J. (1993). What do inventories of students’ learning processes really measure? A theoretical review and clarification. British Journal of Educational Psychology.

[B9-behavsci-16-00781] Biggs J., Kember D., Leung D. Y. (2001). The revised two-factor study process questionnaire: R-SPQ-2F. British Journal of Educational Psychology.

[B10-behavsci-16-00781] Böheim R., Urdan T., Knogler M., Seidel T. (2020). Student hand-raising as an indicator of behavioral engagement and its role in classroom learning. Contemporary Educational Psychology.

[B11-behavsci-16-00781] Chang K. C., Chang Y. H., Yen C. F., Chen J. S., Chen P. J., Lin C. Y., Griffiths M. D., Potenza M. N., Pakpour A. H. (2022). A longitudinal study of the effects of problematic smartphone use on social functioning among people with schizophrenia: Mediating roles for sleep quality and self-stigma. Journal of Behavioral Addictions.

[B12-behavsci-16-00781] Chen J., Huebner E. S., Tian L. (2020). Longitudinal relations between hope and academic achievement in elementary school students: Behavioral engagement as a mediator. Learning and Individual Differences.

[B13-behavsci-16-00781] Chen W., Gao Y., Ren R., Bi Y., Liao Y. (2025). Socioeconomic status and internet addiction: Double-mediated moderation. BMC Public Health.

[B14-behavsci-16-00781] China Internet Network Information Center (2023). The 5th national survey on internet use among minors.

[B15-behavsci-16-00781] Cortina J. M. (1993). What is coefficient alpha? An examination of theory and applications. Journal of Applied Psychology.

[B16-behavsci-16-00781] Curcio G., Ferrara M., Gennaro L. D. (2006). Sleep loss, learning capacity and academic performance. Sleep Medicine Reviews.

[B17-behavsci-16-00781] Dewald J. F., Meijer A. M., Oort F. J., Kerkhof G. A., Bögels S. M. (2010). The influence of sleep quality, sleep duration and sleepiness on school performance in children and adolescents: A meta-analytic review. Sleep Medicine Reviews.

[B18-behavsci-16-00781] Dunkin M. J., Biddle B. J. (1974). The study of teaching.

[B19-behavsci-16-00781] Durmer J. S., Dinges D. F. (2005). Neurocognitive consequences of sleep deprivation. Seminars in Neurology.

[B20-behavsci-16-00781] Dworak M., Schierl T., Bruns T., Strüder H. K. (2007). Impact of singular excessive computer game and television exposure on sleep patterns and memory performance of school-aged children. Pediatrics.

[B21-behavsci-16-00781] Erceg-Hurn D. M., Mirosevich V. M. (2008). Modern robust statistical methods: An easy way to maximize the accuracy and power of your research. American Psychologist.

[B22-behavsci-16-00781] Ewumi A. M. (2012). Gender and socio-economic status as correlates of students’ academic achievement in senior secondary schools. European Scientific Journal.

[B23-behavsci-16-00781] Fonseca A. G., Genzel L. (2020). Sleep and academic performance: Considering amount, quality and timing. Current Opinion in Behavioral Sciences.

[B24-behavsci-16-00781] Fredricks J. A., McColskey W., Christenson S., Reschly A., Wylie C. (2012). The measurement of student engagement: A comparative analysis of various methods and student self-report instruments. Handbook of research on student engagement.

[B25-behavsci-16-00781] Gasevic D., Jovanovic J., Pardo A., Dawson S. (2017). Detecting learning strategies with analytics: Links with self-reported measures and academic performance. Journal of Learning Analytics.

[B26-behavsci-16-00781] Gentina E., Rowe F. (2020). Effects of materialism on problematic smartphone dependency among adolescents: The role of gender and gratifications. International Journal of Information Management.

[B27-behavsci-16-00781] Gi D., Park Y., Kyung M., Park J. (2016). Mobile phone dependency and its impacts on adolescents’ social and academic behaviors. Computers in Human Behavior.

[B28-behavsci-16-00781] Gilbert S. P., Weaver C. C. (2010). Sleep quality and academic performance in university students: A wake-up call for college psychologists. Journal of College Student Psychotherapy.

[B29-behavsci-16-00781] Gravemeijer K., Stephan M., Julie C., Lin F. L., Ohtani M. (2017). What mathematics education may prepare students for the society of the future?. International Journal of Science and Mathematics Education.

[B30-behavsci-16-00781] Group Special Mobile Association (GSMA) (2023). The state of mobile internet connectivity report 2023.

[B31-behavsci-16-00781] Guo J. P., Yang L. Y., Zhang J., Gan Y. J. (2022). Academic self-concept, perceptions of the learning environment, engagement, and learning outcomes of university students: Relationships and causal ordering. Higher Education.

[B32-behavsci-16-00781] Ha Y. M., Hwang W. J. (2014). Gender differences in internet addiction associated with psychological health indicators among adolescents using a national web-based survey. International Journal of Mental Health and Addiction.

[B33-behavsci-16-00781] Hassan T., Alam M. M., Wahab A., Hawlader M. D. (2020). Prevalence and associated factors of internet addiction among young adults in Bangladesh. Journal of the Egyptian Public Health Association.

[B34-behavsci-16-00781] Hawi N. S., Samaha M. (2016). To excel or not to excel: Strong evidence on the adverse effect of smartphone addiction on academic performance. Computers & Education.

[B35-behavsci-16-00781] Hayes A. F., Coutts J. J. (2020). Use omega rather than Cronbach’s alpha for estimating reliability. Communication Methods and Measures.

[B36-behavsci-16-00781] Horwood S., Anglim J. (2019). Problematic smartphone usage and subjective and psychological well-being. Computers in Human Behavior.

[B37-behavsci-16-00781] Hu L. T., Bentler P. M. (1999). Cutoff criteria for fit indexes in covariance structure analysis: Conventional criteria versus new alternatives. Structural Equation Modeling.

[B38-behavsci-16-00781] International Telecommunication Union (ITU) (2023). Facts and figures 2023.

[B39-behavsci-16-00781] Kahneman D. (1973). Attention and effort.

[B40-behavsci-16-00781] Kates A. W., Wu H., Coryn C. L. (2018). The effects of mobile phone use on academic performance: A meta-analysis. Computers & Education.

[B41-behavsci-16-00781] Kaya F., Bostancı Daştan N., Durar E. (2021). Smart phone usage, sleep quality and depression in university students. International Journal of Social Psychiatry.

[B42-behavsci-16-00781] Kim D., Lee Y., Lee J., Nam J. K., Chung Y. (2014). Development of Korean smartphone addiction proneness scale for youth. PLoS ONE.

[B43-behavsci-16-00781] Kim S. Y., Han S., Park E. J., Yoo H. J., Park D., Suh S., Shin Y. M. (2020). The relationship between smartphone overuse and sleep in younger children: A prospective cohort study. Journal of Clinical Sleep Medicine.

[B44-behavsci-16-00781] Kong F., Qin J., Huang B., Zhang H., Lei L. (2020). The effect of social anxiety on mobile phone dependence among Chinese adolescents: A moderated mediation model. Children and Youth Services Review.

[B45-behavsci-16-00781] Lai X., Huang S., Zhang C., Tang B., Zhang M., Zhu C., Wang Y. (2020). The association between smartphone addiction, interpersonal relationships, subjective well-being, and school identity among primary and middle school students. Chinese Journal of School Health.

[B46-behavsci-16-00781] Lane H. Y., Chang C. J., Huang C. L., Chang Y. H. (2021). An investigation into smartphone addiction with personality and sleep quality among university students. International Journal of Environmental Research and Public Health.

[B47-behavsci-16-00781] Lee J., Cho B., Kim Y., Noh J., Chen G., Kumar V., Kinshuk, Huang R., Kong S. (2015). Smartphone addiction in university students and its implication for learning. Emerging issues in smart learning.

[B48-behavsci-16-00781] Lee M. H., Liang J. C., Wu Y. T., Chiou G. L., Hsu C. Y., Wang C. Y., Tsai C. C. (2020). High school students’ conceptions of science laboratory learning, perceptions of the science laboratory environment, and academic self-efficacy in science learning. International Journal of Science and Mathematics Education.

[B49-behavsci-16-00781] Lei H., Cui Y., Zhou W. (2018). Relationships between student engagement and academic achievement: A meta-analysis. Social Behavior and Personality.

[B50-behavsci-16-00781] Li H., Zhu S., Wu D., Yang H. H., Guo Q. (2023). Impact of information literacy, self-directed learning skills, and academic emotions on high school students’ online learning engagement: A structural equation modeling analysis. Education and Information Technologies.

[B51-behavsci-16-00781] Li L., Gao H., Xu Y. (2020). The mediating and buffering effect of academic self-efficacy on the relationship between smartphone addiction and academic procrastination. Computers & Education.

[B52-behavsci-16-00781] Li L., Mei S., Niu Z., Song Y. (2016). Loneliness and sleep quality in university students: Mediator of smartphone addiction and moderator of gender. Chinese Journal of Clinical Psychology.

[B53-behavsci-16-00781] Li Y., Zhang X., Lu F., Zhang Q., Wang Y. (2014). Internet addiction among elementary and middle school students in China: A nationally representative sample study. Cyberpsychology, Behavior, and Social Networking.

[B54-behavsci-16-00781] Liu Q., Du X., Zhao S., Liu J., Cai J. (2019). The role of memorization in students’ self-reported mathematics learning: A large-scale study of Chinese eighth-grade students. Asia Pacific Education Review.

[B55-behavsci-16-00781] Lo J. C., Ong J. L., Leong R. L., Gooley J. J., Chee M. W. (2016). Cognitive performance, sleepiness, and mood in partially sleep deprived adolescents: The need for sleep study. Sleep.

[B56-behavsci-16-00781] Mari E., Biondi S., Varchetta M., Cricenti C., Fraschetti A., Pizzo A. (2023). Gender differences in internet addiction: A study on variables related to its possible development. Computers in Human Behavior Reports.

[B57-behavsci-16-00781] McInerney D. M., Cheng R. W. Y., Mok M. M. C., Lam A. K. H. (2012). Academic self-concept and learning strategies direction of effect on student academic achievement. Journal of Advanced Academics.

[B58-behavsci-16-00781] Nelson A. (2018). Effects of stress, sleep hygiene, and exercise on academic engagement in undergraduate students. Doctoral dissertation.

[B59-behavsci-16-00781] Ng H. T. H., Zhang C. Q., Phipps D., Zhang R., Hamilton K. (2022). Effects of anxiety and sleep on academic engagement among university students. Australian Psychologist.

[B60-behavsci-16-00781] Okano K., Kaczmarzyk J. R., Dave N., Gabrieli J. D., Grossman J. C. (2019). Sleep quality, duration, and consistency are associated with better academic performance in college students. npj Science of Learning.

[B61-behavsci-16-00781] Organisation for Economic Cooperation and Development [OECD] (2017). PISA 2015 technical report.

[B62-behavsci-16-00781] Paterna A., Alcaraz-Ibáñez M., Aguilar-Parra J. M., Salavera C., Demetrovics Z., Griffiths M. D. (2024). Problematic smartphone use and academic achievement: A systematic review and meta-analysis. Journal of Behavioral Addictions.

[B63-behavsci-16-00781] Peris M., de la Barrera U., Schoeps K., Montoya-Castilla I. (2020). Psychological risk factors that predict social networking and internet addiction in adolescents. International Journal of Environmental Research and Public Health.

[B64-behavsci-16-00781] Podsakoff P. M., MacKenzie S. B., Lee J. Y., Podsakoff N. P. (2003). Common method biases in behavioral research: A critical review of the literature and recommended remedies. Journal of Applied Psychology.

[B65-behavsci-16-00781] Rathakrishnan B., Bikar Singh S. S., Kamaluddin M. R., Yahaya A., Mohd Nasir M. A., Ibrahim F., Ab Rahman Z. (2021). Smartphone addiction and sleep quality on academic performance of university students: An exploratory research. International Journal of Environmental Research and Public Health.

[B66-behavsci-16-00781] Sapci O., Elhai J. D., Amialchuk A., Montag C. (2021). The relationship between smartphone use and students’ academic performance. Learning and Individual Differences.

[B67-behavsci-16-00781] Sohn S. Y., Krasnoff L., Rees P., Kalk N. J., Carter B. (2021). The association between smartphone addiction and sleep: A UK cross-sectional study of young adults. Frontiers in Psychiatry.

[B68-behavsci-16-00781] Sun R. Q., Sun G. F., Ye J. H. (2023). The effects of online game addiction on reduced academic achievement motivation among Chinese college students: The mediating role of learning engagement. Frontiers in Psychology.

[B69-behavsci-16-00781] Sun X., Duan C., Yao L., Zhang Y., Chinyani T., Niu G. (2021). Socioeconomic status and social networking site addiction among children and adolescents: Examining the roles of parents’ active mediation and ICT attitudes. Computers & Education.

[B70-behavsci-16-00781] Tehseen S., Ramayah T., Sajilan S. (2017). Testing and controlling for common method variance: A review of available methods. Journal of Management Sciences.

[B71-behavsci-16-00781] Urbanova L. B., Holubcikova J., Madarasova Geckova A., Reijneveld S. A., van Dijk J. P. (2019). Does life satisfaction mediate the association between socioeconomic status and excessive internet use?. International Journal of Environmental Research and Public Health.

[B72-behavsci-16-00781] Wang L., Peng F., Song N. (2022). The impact of students’ mathematical attitudes on intentions, behavioral engagement, and mathematical performance in the China’s context. Frontiers in Psychology.

[B73-behavsci-16-00781] Wang M. T., Fredricks J. A., Ye F., Hofkens T. L., Linn J. S. (2016). The math and science engagement scales: Scale development, validation, and psychometric properties. Learning and Instruction.

[B74-behavsci-16-00781] Watkins M. W. (2017). The reliability of multidimensional neuropsychological measures: From alpha to omega. The Clinical Neuropsychologist.

[B75-behavsci-16-00781] Wong N. Y., Lam C. C., Kong Q. P. (2003). The relationship between student engagement and learning outcome in mathematics. Curriculum and Teaching.

[B76-behavsci-16-00781] Wood A. W., Loughran S. P., Stough C. (2006). Does evening exposure to mobile phone radiation affect subsequent melatonin production?. International Journal of Radiation Biology.

[B77-behavsci-16-00781] Xie W., Berry A., Lustig C., Deldin P., Zhang W. (2019). Poor sleep quality and compromised visual working memory capacity. Journal of the International Neuropsychological Society.

[B78-behavsci-16-00781] Xie X., Dong Y., Wang J. (2018). Sleep quality as a mediator of problematic smartphone use and clinical health symptoms. Journal of Behavioral Addictions.

[B79-behavsci-16-00781] Yang J., Fu X., Liao X., Li Y. (2020). Association of problematic smartphone use with poor sleep quality, depression, and anxiety: A systematic review and meta-analysis. Psychiatry Research.

[B80-behavsci-16-00781] Yang S. Y., Lin C. Y., Huang Y. C., Chang J. H. (2018). Gender differences in the association of smartphone use with the vitality and mental health of adolescent students. Journal of American College Health.

[B81-behavsci-16-00781] Yi H., Shin K., Shin C. (2006). Development of the sleep quality scale. Journal of Sleep Research.

[B82-behavsci-16-00781] Zhang Y., Qin X., Ren P. (2018). Adolescents’ academic engagement mediates the association between Internet addiction and academic achievement: The moderating effect of classroom achievement norm. Computers in Human Behavior.

[B83-behavsci-16-00781] Zhen R., Li L., Ding Y., Hong W., Liu R. D. (2020). How does mobile phone dependency impair academic engagement among Chinese left-behind children?. Children and Youth Services Review.

[B84-behavsci-16-00781] Zhou D., Du X. F., Hau K. T., Luo H. F., Feng P. T., Liu J. (2020a). Teacher-student relationship and mathematical problem-solving ability: Mediating roles of self-efficacy and mathematical anxiety. Educational Psychology.

[B85-behavsci-16-00781] Zhou D., Liu J., Liu J. (2020b). The effect of problematic Internet use on mathematics achievement: The mediating role of self-efficacy and the moderating role of teacher-student relationships. Children and Youth Services Review.

[B86-behavsci-16-00781] Zhou D., Liu J., Liu J. (2021). On the different effects of teacher-student rapport on urban and rural students’ math learning in China: An empirical study. Psychology in the Schools.

[B87-behavsci-16-00781] Zhou D., Liu J., Wang T., Liu J., Li G. (2022). Relationships among problematic smartphone use, mathematics anxiety, learning interest, and achievement: A multiple mediation model. Computers in Human Behavior.

